# Understanding student perceptions of social computing and online tools to enhance learning

**DOI:** 10.1371/journal.pone.0276490

**Published:** 2022-10-27

**Authors:** Semiyu Adejare Deribigbe, Wafa Barhoumi Hamdi, Khadeegha Alzouebi, William Frick, Assad Asil Companioni

**Affiliations:** 1 College of Arts, Humanities & Social Sciences and Institute of Leadership in Higher Education, University of Sharjah, Sharjah, UAE; 2 College of Arts, Humanities & Social Sciences, University of Sharjah, Sharjah, UAE; 3 School of e-Education, Hamdan Bin Mohamed Smart University, Dubai, UAE; 4 Jeanine Rainbolt College of Education, University of Oklahoma, Norman, Oklahoma, United States of America; 5 College of Arts, Humanities & Social Sciences, University of Sharjah, Sharjah, UAE; King Abdulaziz University, SAUDI ARABIA

## Abstract

Social computing software and online tools are gaining credence in teaching and learning spaces, including higher education contexts. However, the adoption of social computing software does not automatically translate into effective teaching and learning if students’ views and needs are not considered along with course learning outcomes. Thus, this study was conducted to explore students’ perspectives and preferences for social computing software and online tools in a university elective course. We employed quantitative and qualitative approaches to understand students’ shared and nuanced thoughts about social computing applications in the study. A questionnaire with quantitative and open-ended qualitative questions was used to collect data. Data were analyzed using descriptive statistics and an inductive thematic analysis approach. Our findings indicated mixed students’ viewpoints, with some tools reported as highly beneficial while others were less beneficial. For instance, students valued asynchronous sessions, assignment feedback, online discussion, videos, and gamification but reported less interest in phones, journals, icons, and blogs. Students’ specializations also appeared to influence their choice of tools. Those from Arts, Humanities, and Social Sciences expressed a slightly different preference than their comparts from Medicine and Health Sciences. Drawing on the findings, we discuss the implications for effective teaching and learning using social computing software, focusing on essential stakeholders. For instance, instructors must regularly conduct diagnostic feedback to determine appropriate tools that can effectively customize students’ learning.

## Introduction

In the pre-COVID-19 era, technology gained credence as a tool for effective teaching and learning due to its dynamic features for assisting with different students’ needs and fostering inclusivity. Due to technology’s capabilities and the need to keep pace with societal needs, it was reasonably predicted that the educational process would feature more online endeavors using social computing and online tools to enhance students’ learning [1]. Besides, the sudden transition to fully online teaching and learning platforms due to the COVID-19 pandemic has accelerated and increased the use of social computing software and active learning strategies [2,3]. In strengthening activity-based and impactful teaching strategies, many educators shifted from instructors to facilitators [4], guiding students to take ownership of the learning process using various social computing software. Correspondingly, social computing software gained more prominence as face-to-face interactions reduced while simultaneously increasing the need for alternative means to engage and foster students’ academic and psychological well-being. Scholars contend that contemporary society is experiencing a fast-paced development and deployment of computing tools in all spectrums, including the educational sector [5]. Building a new set of skills, including technology and digital-related transferrable skills, is an essential obligation of educational institutions to prepare students for a productive and functional society [6].

However, researchers warn that using online tools and social computing software will not automatically translate into effective online teaching and learning experiences due to inherent challenges associated with the modality [7]. For instance, technological landscapes and functionalities are constantly changing, along with the characteristics and needs of users. Consequently, this demands that educators are able and willing to update content regularly [8]. It is also stated that non-user-friendly services may hamper the functionality of social computing applications and create privacy issues [9]. Similarly, inadequate facilitation knowledge on how to effectively use social computing software has the potential to elicit technical anxiety associated with their use [10]. Additionally, educators express concerns about the management aspect of innovative social computing tools [11], which can adversely impact the online teaching and learning process [12].

Negative impressions about online education may affect the online learning process involving social computing tools. A research report on student attitudes towards online education during the COVID-19 pandemic indicates that students do not believe online education quality is at par with traditional face-to-face pedagogy [13]. This view held by some students is likely to affect their commitment and engagement in learning when interfacing these social computing software programs and tools in their courses. These perceptions and attitudes indicate that effective pedagogical strategies and technology depend on a certain degree of knowledge about students’ needs, preferences, and treasured values. Thus, understanding students’ perceptions of social computing software and online tools are essential for supporting the educational process. Perhaps, this explains why the literature suggests the need for educators to track students’ thinking about their learning processes (in the form of indirect assessment leading to measurement about how learning has occurred) as online education gains more prominence and more classes transition to online milieus [14].

Furthermore, it is argued that learning facilitators must continually explore frameworks and actions to enhance and sustain effective online pedagogy [7,15]. Doing this, and having adequate knowledge about students’ preferences, will assist educators in effectively deploying social computing tools using appropriate pedagogies for stimulating students’ curiosity and motivation, thereby positively impacting their learning. As reported, understanding users’ needs and the capacity to utilize digital tools for achieving desired outcomes is essential for the effective design and deployment of devices [8]. The literature also indicates that effective digitization goes beyond technological tool changes and utilization; efforts must focus on the relationships between the devices and use factors for effective deployment [6]. Another study indicates a dearth of studies on technology applications in educational processes despite young people’s passion for technology usage in the Arab world [16]. These revelations indicate the need for educators in the region and elsewhere globally to intensify efforts at developing an understanding of students’ preferences and feedback on social computing tools in support of their learning.

Against the aforementioned background, we undertook this study to explore students’ views on social computing software and the online tools used to facilitate learning in an undergraduate university elective course. We aligned our thoughts with the literature, indicating the need for an ongoing revision in pedagogical approaches and the application of social computing tools for effective contextualized educational processes. Drawing on the study’s outcomes, we hope to offer insight into evidence-based strategies to enhance the use of online educational software and tools for effective teaching and learning experiences. We also contend that findings from the study could provide valuable student-centric insights to instructional designers and course developers for strengthening their online course redesign processes. In the next section, we present the conceptual framework, after which we discuss the research methods we employed. In the last three portions, we share the findings, discuss the results along with the extant literature and articulate our conclusion and recommendations.

### Conceptual framework

Social computing involves the application of computing software to support social relationships [17]. It is also described as human-technology interaction to foster social engagement, collaboration, and interactions [9,18,19]. Researchers argue that social computing software connotes the online technological tools that support collaborative and interactive engagements, such as blogging, social networking, wikis, and podcasting [20]. Perhaps, this explains why some literature suggests that e-learning platforms and social computing software may assist students in developing self-study skills and even enhance academic achievement compared to traditional teacher-centered strategies [21].

Literature also indicates that social computing software offers the opportunity for learners and educators to collaborate, communicate and share content [20]. Notably, the flexible, editable, and multi-media capabilities of e-courses supported by social computing software assist students in learning regardless of where they dwell or their educational level [22,23]. Not surprisingly, the effective use of social computing and online learning tools is reported to enhance students’ learning, offering them the chance to learn from one another and strengthen the knowledge-building process [4,24]. Social computing software such as discussion forums, wikis, blogs, and learning management systems (LMS) provide “on-the-go learning experiences” for students [2].

The use of social computing software and online tools are grounded in the constructivist and knowledge-building community frameworks that offer the opportunity for collaboration, co-construction of knowledge, active engagement, and scaffolding. From the constructivist strand, social computing software aids personal interpretations, knowledge construction, and reflective practices [25,26]. Using social computing software can also align with a knowledge-building community’s goals and principles. As explained, a knowledge-building community holds that learning occurs within a group where individuals collaborate to strengthen knowledge development [27]. Educators need to rethink the teaching and learning process involving today’s students (digital natives), enabling them to contribute effectively in dynamic, technology-enabled work environments [6].

**Fig 1:**
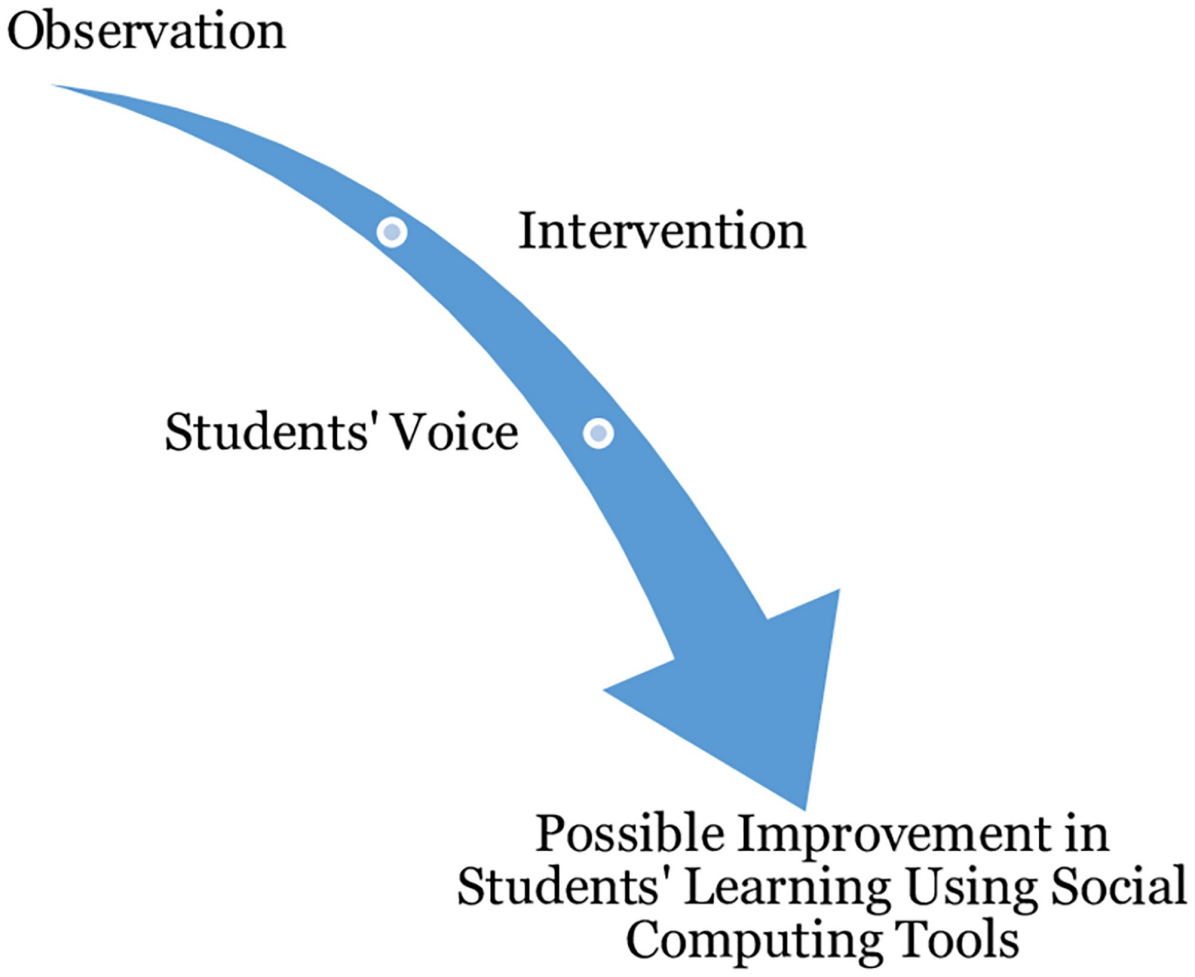
Research framework.

However, we acknowledge that social computing applications might not achieve desired engagement and learning goals due to inherent challenges. For instance, the tools can be counter-productive if not well-managed, and students may not be able to use them appropriately due to inadequate knowledge and anxiety associated with the platforms [16,28,29]. A well-documented measure for reducing or alleviating the challenges is to obtain students’ feedback about the kind of support they may need to take advantage of the tools [4,16,22]. In alignment with this, we agree with other scholars about the need to consider students’ thoughts on what they find beneficial and problematic. Doing this will enable us to evaluate those technological features that may require reconfiguration for more effective future deployment and utilization. Following this model, we believe, will improve students’ learning experiences using social computing software. Fig 1 provides a graphical representation of the staged framework trajectory.

In guiding the data collection process, we crafted and explored the following research questions:

1. What social computing software and tools do students consider the most beneficial in the context of their course?
2. Are there differences in students’ preferences for the social computing software and tools used in the course based on their specializations?
3. What social computing software and tools do students consider essential for planning and delivering the course in the future?
4. Are there differences in students’ preference for the social computing software used and tools to be considered for effective delivery of the course in the future based on their specializations?

## Methods

### Design

We employed a descriptive research design for this study as it offers the opportunity to define existing conditions or relationships between incidents [30]. In using this method, we sought to portray students’ perceptions of social computing and online tools used in different contexts and the possible implications of their views for the future design and delivery of coursework. We conducted the study using a mixed-methods approach, combining and integrating quantitative and qualitative data. In the quantitative phase, we captured the shared views of the students related to their experience with social computing software and online learning tools. On the other hand, the qualitative strategy allowed us to document the thoughts of the students clarifying their choices of options at the quantitative stage. This approach allowed us to gain a deeper insight into issues related to student preferences for the social computing software and online learning tools adopted and their impact on in their classes. We adopted this method to enhance our understanding of complex issues and phenomena [31] and lend credibility to research outcomes by triangulating different data sets [32].

### Instrumentation

We designed an online questionnaire to collect data through a reflection on the social computing tools found beneficial to student learning in the literature which is currently being used in university coursework. We decided to use a questionnaire to elicit responses and information from as many students as possible.

The questionnaire had three elements–demographic information, quantitative, and qualitative questions (See appendix). The quantitative part had closed-ended questions offering the respondents limited indicated selections presented in a similarly structured manner [33,34]. Students were asked to select and rank social computing tools found beneficial in their classes and those to be considered in future planning for the course delivery. On the other hand, the open-ended questions allowed students to clarify the reasons for their choices in the quantitative section, drawing on their peculiar understanding and experience, thereby providing room for divergent and nuanced thoughts. Using this approach allowed us to substantiate students’ views about course planning and delivery tools within comparable future circumstances.

### Context and data collection procedure

We conducted this study in the context of an undergraduate elective, or general education (GNED) taught synchronously online and supported with social computing tools in two classes at a semi-private University in the United Arab Emirates (UAE). The course complements core courses at the university, and it is taken by students across the colleges in the university. A total of 113 students were in the two classes. In administering the questionnaire, we shared an electronic link with all students in two university elective classes during the last week of the Spring 2021 semester and sent two reminders to the students. We accessed the students’ responses after their results were released, and we found that 64 out of the 113 students completed the questionnaire, accounting for a 57% participation response rate. We then grouped them into two categories based on their participation rates, with those in Arts, Humanities, and Social Sciences oriented courses accounting for 22% and 52% in Medical and Health Sciences. We acknowledge that the response rate is a limitation to the study, and as a result generalizations based on these findings is very challenging. Nevertheless, we do believe that the results provide valuable insights and foundational conceptions to support future large-scale studies.

### Validity and trustworthiness

In the pre-data collection stage, we validated the questionnaire through an iterative and collaborative process of reviewing and revising the questionnaire’s contents, drawing on feedback from colleagues and students who read the initial draft. Later, we used Cronbach’s Alpha (α) to determine the internal consistency reliability of the data collected. The Cronbach’s Alpha approach holds that all items must consistently measure the same domain. A questionnaire is considered internally consistent (reliable) when there is a high correlation score of considered items [35]. As explained, the α value ranges from 0.0 to 1.0; the closer α is to 0.0, the less consistent the items and, therefore, less reliable; the minimum requirement for a questionnaire to be reliable is a value of 0.7 [35]. The computed α for the data in this study has a value of 0.879, which indicates a sufficiently reliable result.

To profoundly investigate the reliability of the results by each item, the α was computed after eliminating each item individually. This method assisted in investigating the effect of the items on the reliability of the results. The Cronbach’s Alpha results are shown in Table 1. The α value was not significantly affected by the removal of any item, which indicates the good correlation between results obtained and items rating. The lowest value of α (0.866) was obtained when the item ‘Email’ was deleted.

**Table 1:** Results of Cronbach’s Alpha (α) after e deletion of each item.

Item	α if Item Deleted
Online discussion forum	0.871
Blogs	0.876
Journals	0.879
Assignment feedback	0.867
Online class (through Collaborate)	0.867
Chat box in Collaborate	0.870
Icons in Collaborate	0.867
Video clips	0.870
Online class group discussion	0.872
Gamification (Kahoot Game)	0.879
Online office hour (through Collaborate)	0.867
Email	0.866
Phone	0.869
MS Team	0.871
Announcement (on Blackboard)	0.867

Moreover, 0.5 scorings’ standard deviations were added as error bars (for Figs 6, 10 and 11) to investigate the validity of the results at higher and lower scatters [36]. It can be seen that the conclusions would be reasonably similar considering higher or lower 0.5 standard deviations, ensuring the validity of the results at the highest and lowest scatter.

### Data analysis

We analyzed the data collected in two parts. In the first instance, we analyzed the quantitative data (questions 1 and 3) descriptively using the SPSS version 27, highlighting and ranking students’ preferences in percentages, mean scores, and standard deviations. On the other hand, we used NVivo version 12 to code the open-ended qualitative data (questions 2 and 4) through an inductive analysis approach which “research findings to emerge from the frequent, dominant, or significant themes inherent in raw data” [37, p.238]. In doing this, we used NVivo to generate codes from the extracts of the respondents’ views. We later compared the codes and categorized them under themes depicting the prevailing ideas [38,39]. Lastly, we presented the data in figures and integrated them to discuss the findings along with the extant literature highlighting the study’s implications for stakeholders, such as faculty, instructional designers, and university administration.

### Ethical considerations

We ensured compliance with ethical standards in following these research procedures. In the research context, all research proposals and activities are reviewed and ethically cleared by the College’s Research and Scientific committee at the initial stage. Researchers are advised to make modifications and address any ethical concerns before proceeding if necessary. In our case, the committee gave the ethical clearance at the beginning of the Spring semester in the 2020/2021 academic session without raising any concerns, as participants were not at risk if they chose or declined to participate. Further, there was no need to seek parents’ consent for the undergraduate students involved in the study. Additionally, we explained the purpose of the research, clarified that it was their right to participate or not to participate in the study, and assured them that their participation was confidential. Students were asked to acknowledge they understood the essence of the survey and were willing to give their consent to complete the questionnaire anonymously.

## Results

In this section, we present our findings in relation to the study’s research questions.

### 4.1 Research Question 1

What are the social computing software and tools considered to be the most beneficial to students in the course?

**Fig 2:**
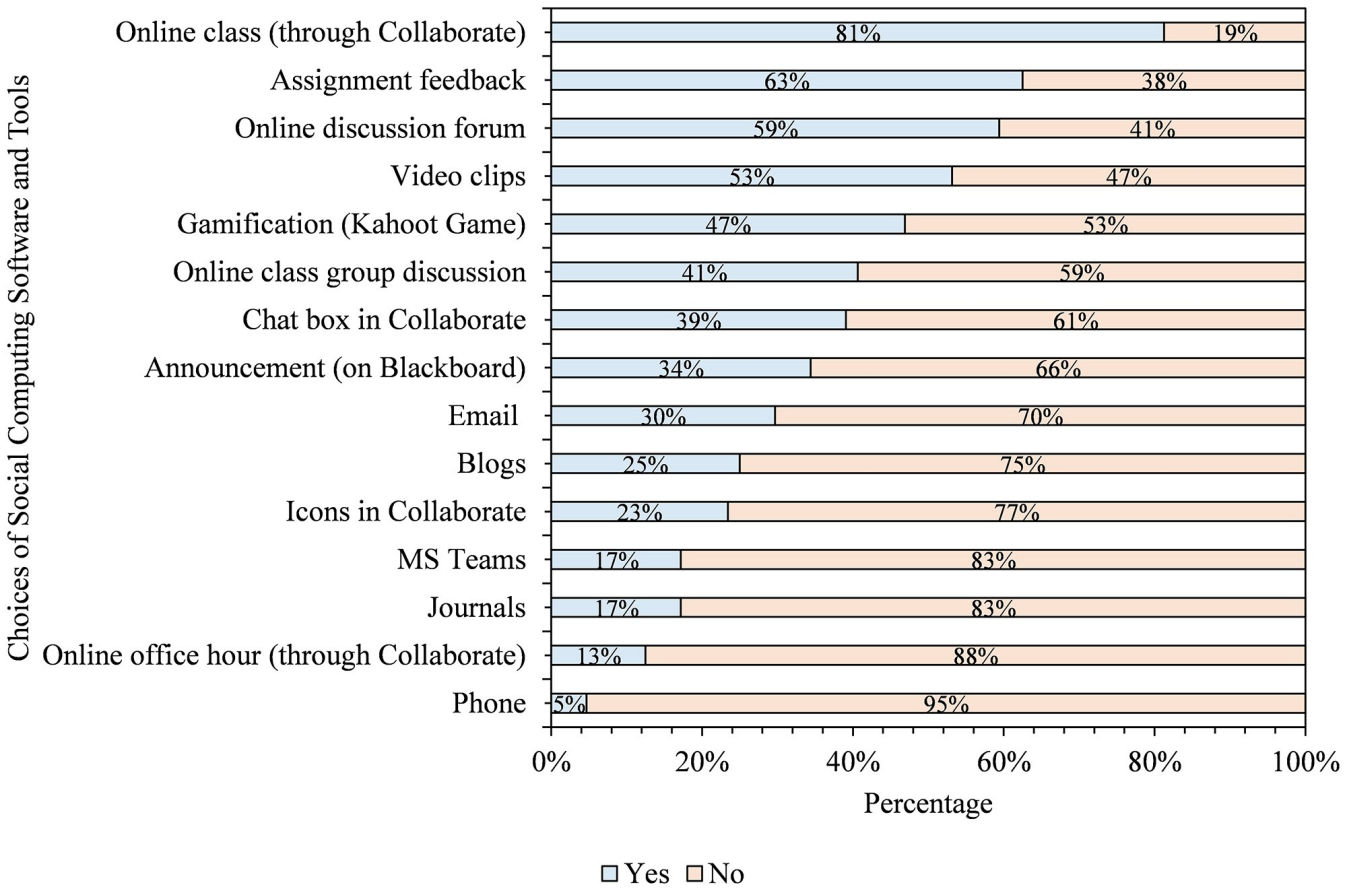
Students’ choices of social computing software and tools that helped them to learn in the course (n = 64).

We asked students to indicate the social computing software most beneficial to them in answering the question, drawing on their experience. We then calculate the percentage of students’ response rate to each tool used in the course. As shown in Fig 2, the first five tools considered most helpful in the course are online class (84%), assignment feedback (63%), online discussion forum (59%), video clips (53%), and gamification (47%). On the other hand, the five least beneficial tools were phone (5%), MS Teams (17%), journals (17%), icons in Blackboard collaborate (Bb) (23%), and blogs (25%).

**Fig 3:**
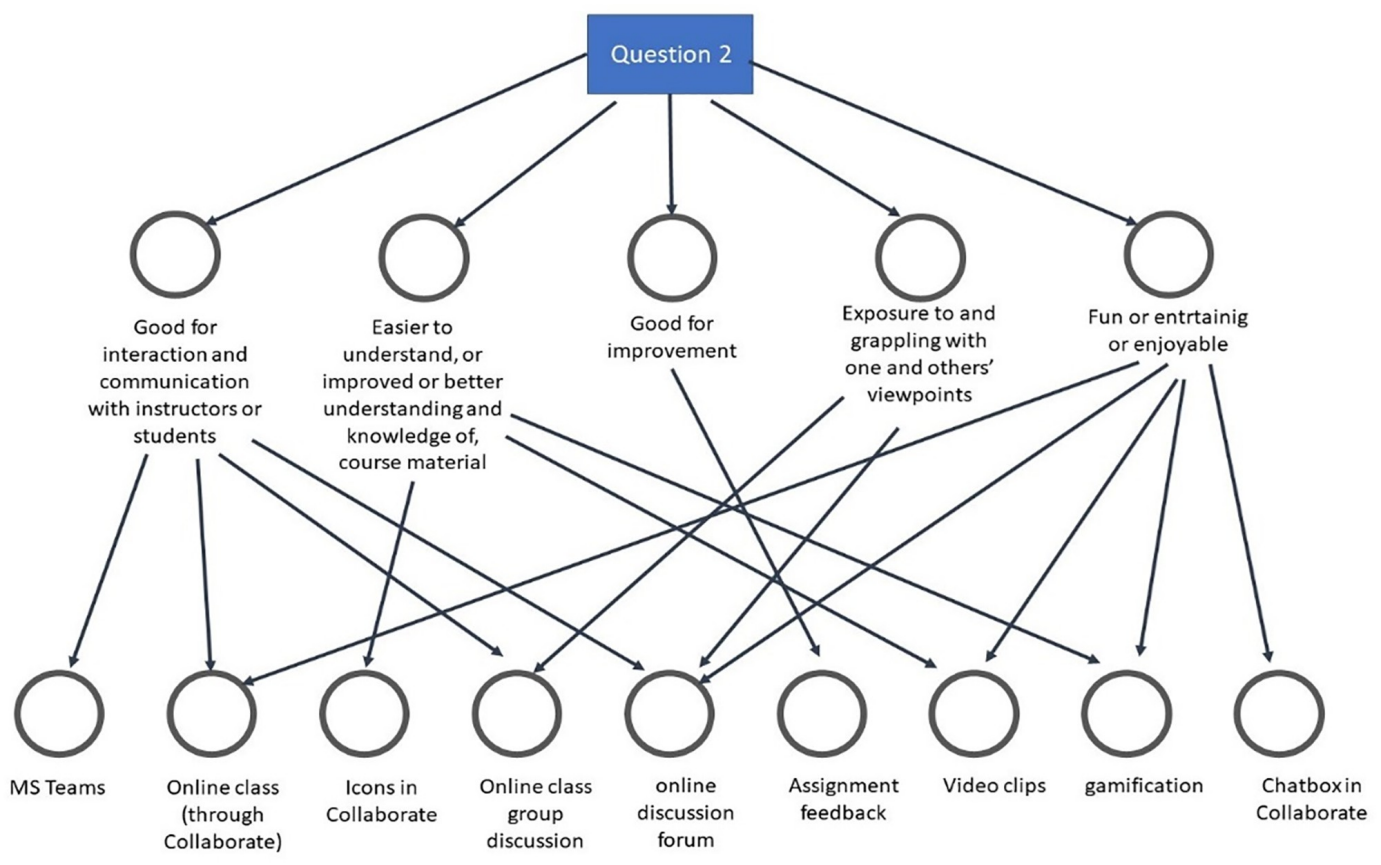
Reasons for students’ choices of social computing software and tools used in the course.

As Fig 2 showed, students indicated that some tools are highly beneficial while they considered some less valuable in the course. It is safe to suggest that a well-planned and deployed synchronous online class, assignment feedback, and online discussion can aid students’ learning in similar courses. However, we wanted to understand the reasons behind students’ choices in Fig 2. Therefore, we asked them to clarify why they had chosen the options reported and thematically analysed their responses. Fig 3 shows why students selected the options documented in Fig 2.

From the analyzed first open-ended question in the questionnaire, five themes emerged as reasons why students thought some social computing tools were more beneficial than others. As indicated in Fig 3, students felt the selected useful tools reported in Fig 2 helped them interact and communicate in groups. In particular, they explained that online class group discussion, online discussion forums, and online classes assisted them in engaging and communicating among themselves and with their instructor. Also, students thought the chosen tools helped them better understand concepts explored in the course. For this theme, they identified gamification, video clips, and icons in Bb Collaborate as tools with the potential to assist students in having a better understanding of course content.

Similarly, students explained that assignment feedback assisted them in improving their learning process. Besides, students contended that online discussion forums and online tools exposed them to others’ thinking and helped them demonstrate their knowledge. Lastly, students explained that their choices were because the tools offered them the opportunity to have fun while learning, citing videos, gamification, and the chat box in Bb Collaborate as enjoyable and fun-packed. Even so, we also wanted to know if the sentiments shared above are generalizable to the students across different disciplinary areas. So, we analyzed the data related to four colleges, as seen below.

### 4.2. Research Question 2

Are there differences in students’ preference for the social computing software and tools used in the course based on their specializations?

**Fig 4:**
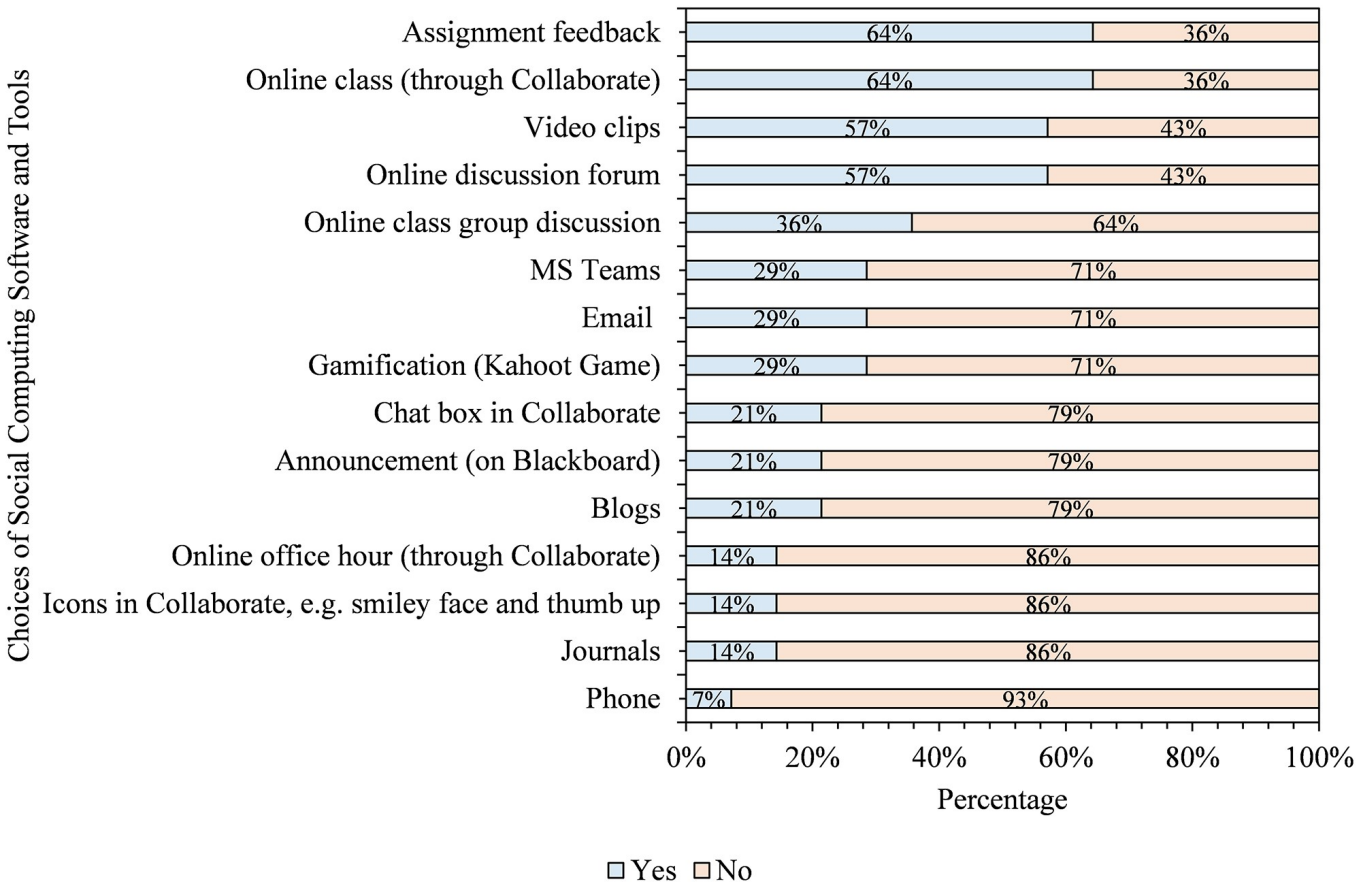
Arts, humanities and social sciences, and business students’ preference for online learning tools used in the course.

In answering this question, we grouped the Colleges of Arts, Humanities and Social Sciences (AHSS) and Business on the one hand. On the other hand, we grouped the Colleges of Health Sciences and Medicine and then compared findings from the two groups. We then calculate the percentage of their responses to each item. Fig 4 shows students’ views from the Colleges of AHSS and Business.

**Fig 5:**
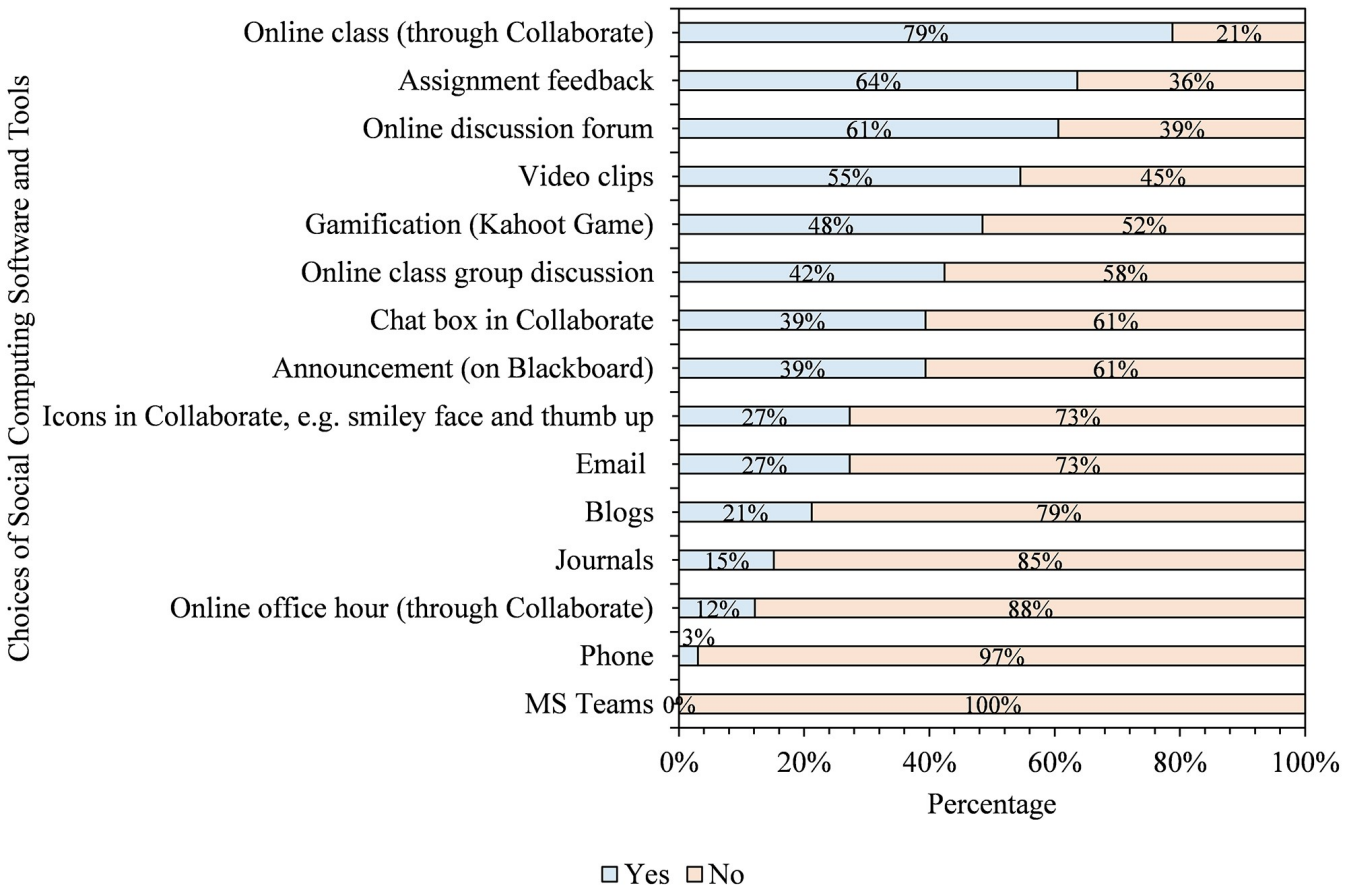
Health sciences and medicine students’ preference for online learning tools in the course.

As shown in Fig 4, AHSS and Business students found online classes (71%), assignment feedback (64%), video clips (57%), and online discussion forums (57%) to be the most beneficial tools in their learning process. On the other hand, they found the phone (7%), online office hours (14%), journals (14%), and icons in Bb Collaborate (14%) less helpful. Facilitators in the fields and GNED courses with diverse students could consider exploring these findings in planning and applying these tools for maximum benefits. To determine whether Health Sciences and Health Sciences students share the same sentiments, we analyzed their responses and choices of social computing tools. Fig 5 shows students’ views on Health Sciences and Medicine regarding the most beneficial means of learning.

As shown in Fig 5, Health Sciences and Medicine students reported online classes (82%), assignment feedback (64%), online discussion forums (61%), and video clips (55%) as their top four tools helpful in their learning process. Conversely, the phone (3%), online office hours (12%), journals (15%), and blogs (21%) are less valuable to the students. From Figs 4 and 5, online classes, assignment feedback, and video clips are common to the two groups, even though they were ranked differently. Also common to the two groups as less useful tools are phones, office hours, journals, and blogs. Thus, considering these findings when planning, designing and delivering similar courses can positively impact the teaching and learning process. That notwithstanding, we also wanted to check if students will recommend the tools they found beneficial or others as those that educators should consider in the future as our third research.

### 4.3. Research Question 3

What are the social computing software and tools that students consider essential for effective teaching and learning in the future?

**Fig 6:**
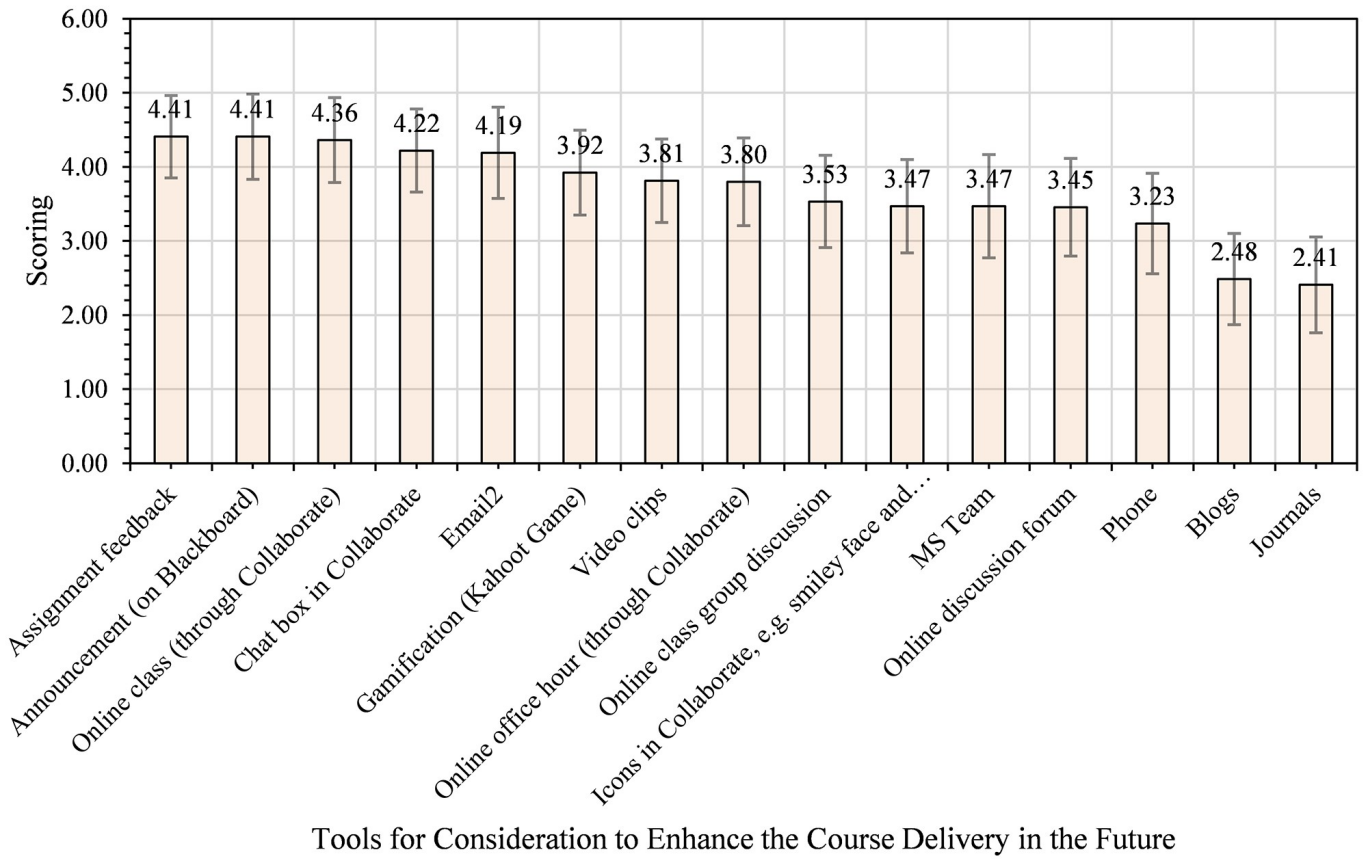
Students’ views on tools for consideration to enhance the course delivery in the future.

In answering the question, we asked students to reflect on their experience and suggest tools for future consideration to enhance teaching and learning in similar contexts. We then calculate the mean score of their responses to each item, showing their view’s strength from 5 downward to 1. Fig 6 shows the tools suggested as essential for enhancing the teaching and learning process in the course in the future.

From Fig 6, students indicated announcement on Bb (4.41), assignment feedback (4.41), online class using Bb Collaborate (4.36), and email (4.19) as essential social computing tools for consideration to enhance the teaching and learning process in the course in the future. At the bottom end are journals (2.41), blogs (2.48), and phones (3.23). As the data showed, announcements on Bb and email appeared in the top spots for the first time for all the students involved in this study. Thus, the two tools also need to be considered when planning to enhance the teaching and learning process in the future using social computing tools.

**Fig 7:**
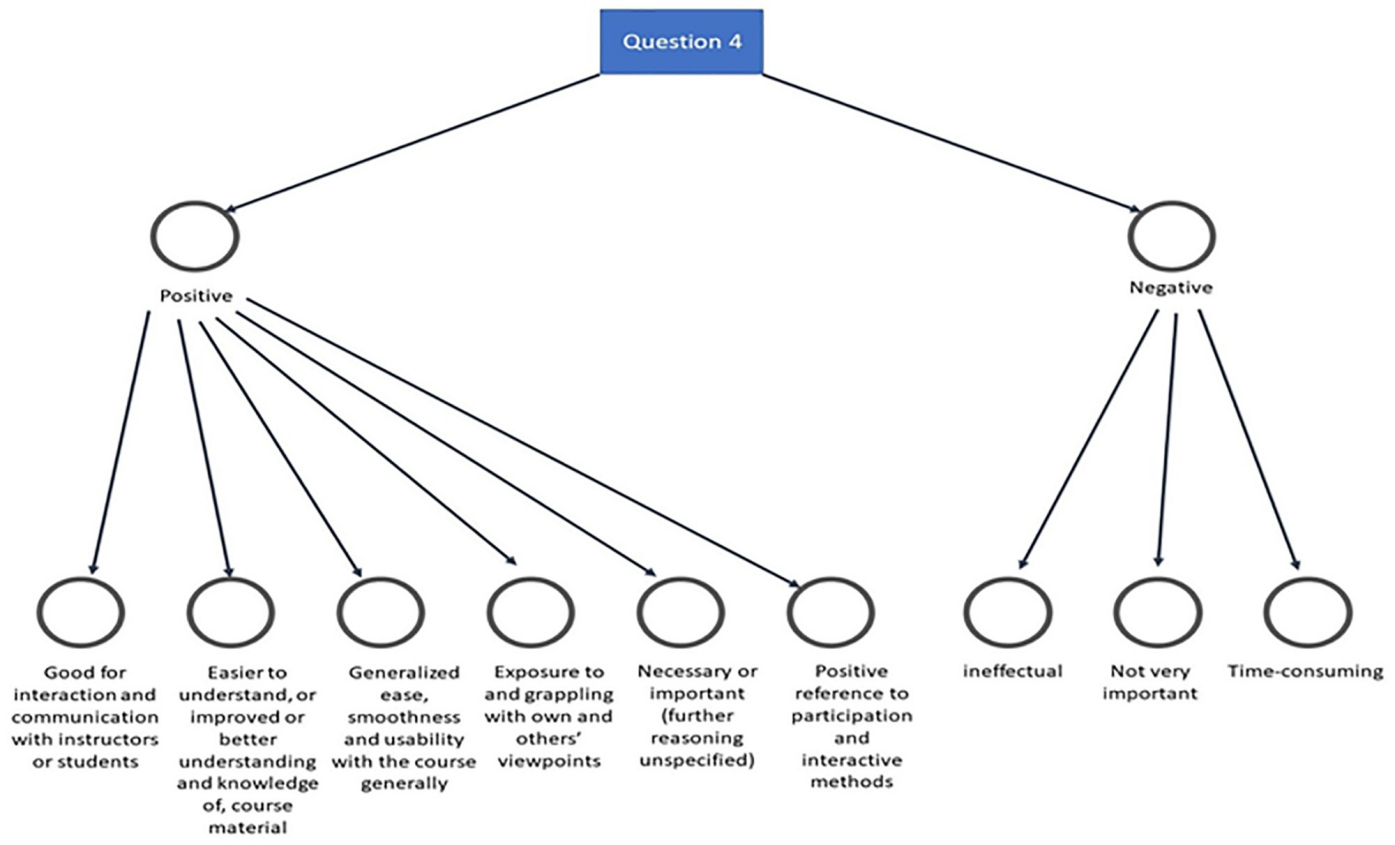
Reasons for students’ choice of tools for enhancing course delivery in the future.

To understand why students have recommended some software over the others, we asked them to give reasons for their selections in the qualitative phase and analysed their views thematically. After analysing their thoughts, we found positive and negative points related to their choices. Fig 7 shows why students subscribed to the need for some software to be considered in the future over the others.

**Fig 8:**
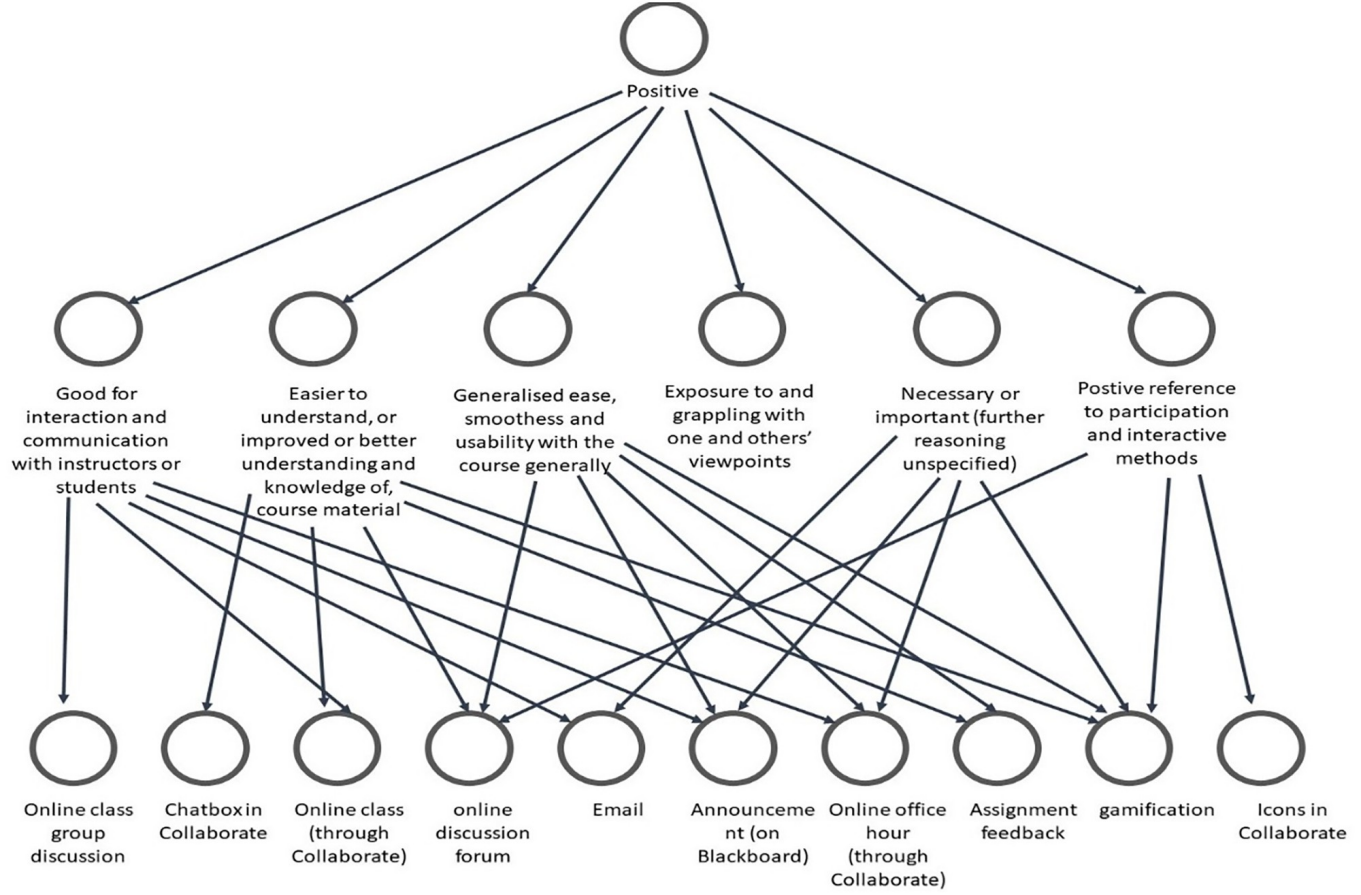
Codes associated with positive themes regarding tools for consideration in the future.

As Fig 7 revealed, students’ expressed positive views about some social computing software and tools because they are suitable for interaction, enhance their understanding, and are easy to use. Conversely, they considered some tools less beneficial because they are ineffectual, unimportant, and time-consuming. Besides, we analysed the positive and negative themes based on the students’ thoughts to expound on the tools recommended for future consideration. Fig 8 shows the associated codes to the positive themes.

**Fig 9:**
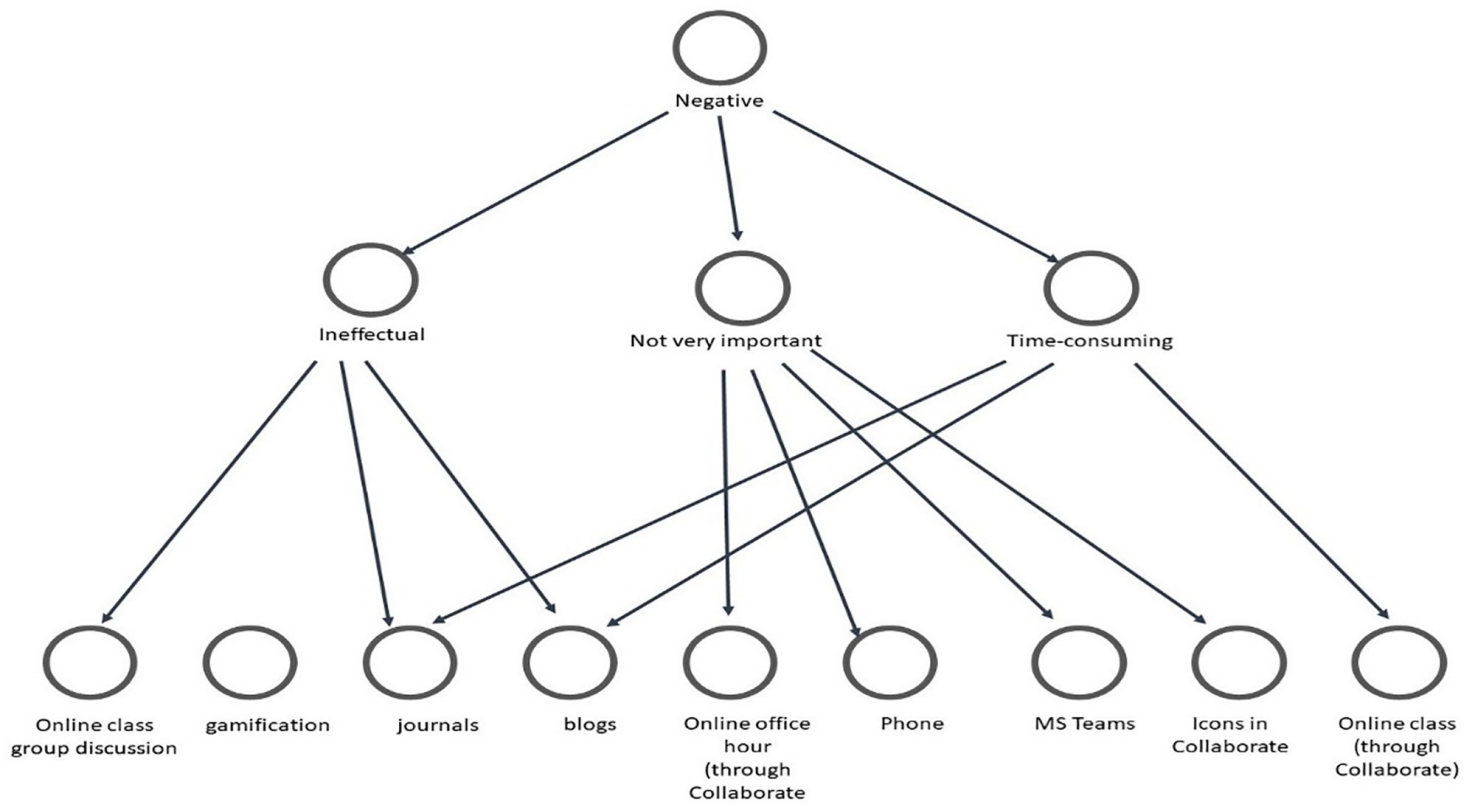
Codes associated with negative themes regarding tools for consideration in the future.

As shown in Fig 8, assignment feedback, gamification, online discussion, online class, and announcement are the dominant tools considered beneficial and worthy of future consideration. This finding reaffirms the vital place of these tools and the need for educators to consider them when similar courses’ planning and delivery. On the other hand, Fig 9 shows students’ views related to negative thoughts about some social computing software and tools.

As shown in Fig 9, students’ negative sentiments about some tools are clarified. However, blogs and journals are those with the most resentment, and educators need to consider this in the future. In addition, we wanted to know if the feelings shared above were generalizable to all the students in this course. Thus, we analyzed and presented the data related to the selected four colleges as follows.

### 4.4. Research Question 4

Are there differences in students’ preference for the social computing software to be considered for effective delivery of the course in the future based on their specializations?

**Fig 10:**
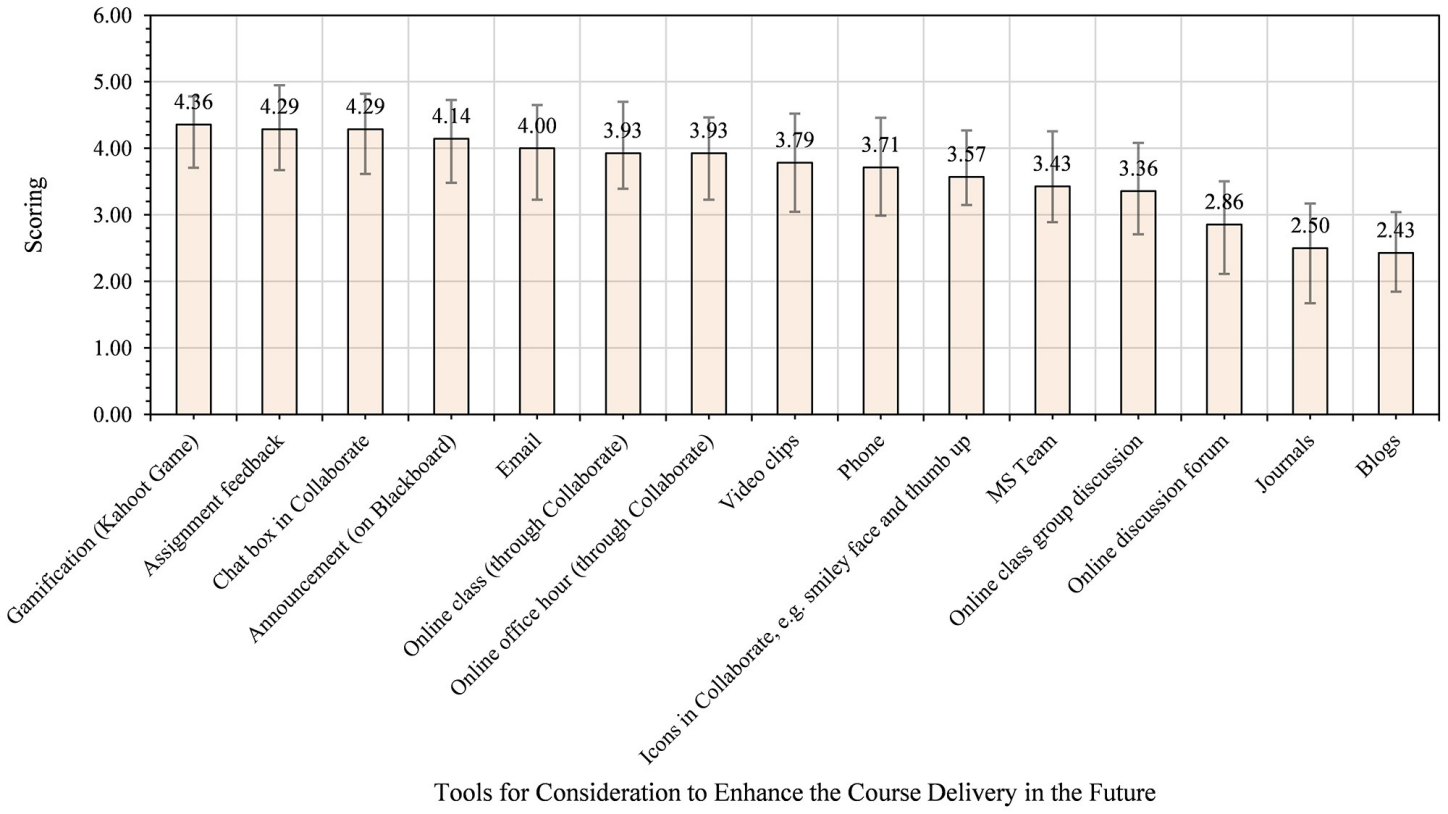
Arts, humanities and social sciences, and business students’ views on tools for enhancing course delivery in the future.

In determining whether students from the two groups shared the same sentiments regarding the tools for effective course delivery in the future, we analyzed the mean scores of their views as presented in the graphs below. Fig 10 shows the perspectives of AHSS and Business students concerning social computing software and tools to enhance teaching and learning in the course in the future.

**Fig 11:**
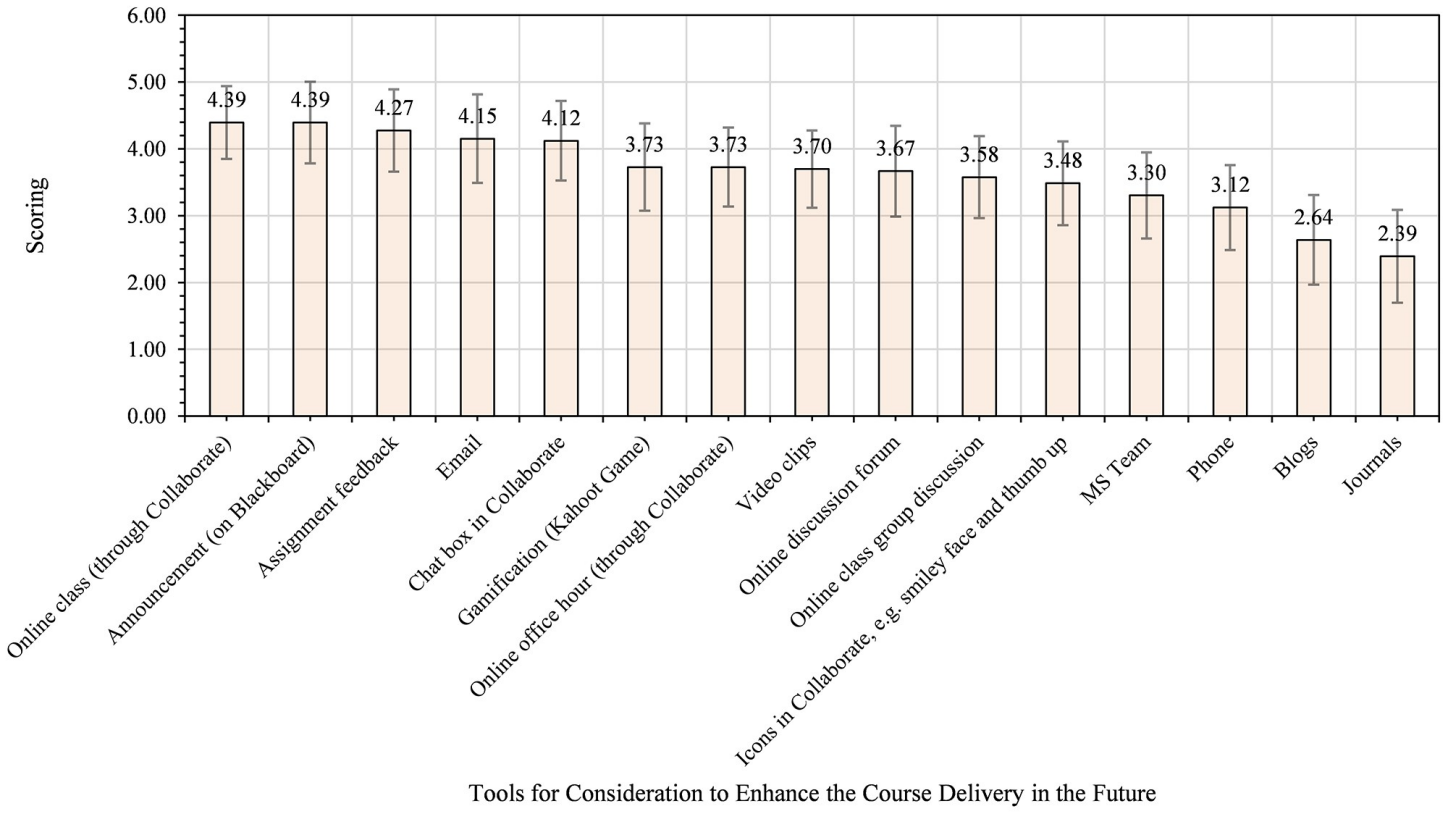
Health sciences and medicine students’ views on tools for enhancing course delivery in the future.

As Fig 10 indicated, students of AHSS and Business identified gamification (4.36), assignment feedback (4.29), chat box in Bb Collaborate (4.29), announcement (4.14), and email (4) as the top five essential tools to enhance course delivery in the future. In Fig 11 below, the views of Health Sciences and Medicine students are presented.

As seen in Fig 11, Health Sciences and Medicine students thought announcement (4.39), online classes (4.39), assignment feedback (4.27), email (4.15), and chat box in Bb Collaborate (4.12) are the top five tools for strengthening course delivery in the future. Drawing on Figs 10 and 11, it is apparent that announcements, online classes, assignment feedback, and email are the four common tools for enhancing teaching and learning in the course in the future. The AHSS and Business students identified gamification as the fifth top tool, while Health Sciences and Medicine chose the online class. These findings suggest that AHSS and Business appreciate more asynchronous activities supported with gamification. Conversely, Health Sciences and Medicine students may need more synchronous sessions to seek clarifications on their class activities. Table 2 presents the synopsis of students’ views and preferences of social computing software and tools.

**Table 2:** A synopsis of students’ views and preference of social computing software.

Views	Social Computing Software and Tools
Highly Beneficial to all Students	Online synchronous class Assignment feedback Online discussion forum Video clips Gamification
Less Beneficial to all Students	Phone MS Teams Journals Icons in Bb LMS Blogs
Highly recommended for Effective Learning by AHSS and Business Students	Gamification Assignment feedback Chat box (in Bb Collaborate) Announcement Email
Highly recommended for Effective Learning by Health Sciences and Medicine Students	Announcement Online classes Assignment feedback Email Chat box

## Discussion

In this study, we investigated different social computing tools used to support students’ learning in two university elective classes within the pandemic timeframe. We considered the endeavor essential so we could learn from students about what worked for them and what we needed to consider for effective course design and delivery in the future. Similar to previous studies [9,21], students found social computing software beneficial and would like educators to consider particular options for effective future teaching and learning. Students indicated that online synchronous class, assignment feedback, online discussion forums, video clips, and gamification assisted learning in the courses. Conversely, they thought phones, MS Teams, journals, icons in Bb Collaborate, and blogs were less beneficial. These findings indicate the need for instructors to strategically select and deploy social computing tools for enhancing students’ collaborative and authentic learning. Indeed, offering students the opportunity to collaborate using appropriate educational tools can foster the development of a knowledge-building community and collaborative knowledge creation [27].

Students may also feel overwhelmed and experience technological anxiety when many tools are used. So, selecting and using a few social computing tools in line with students’ preferences should top the priority lists of instructors aiming to enhance students’ learning in an educational process grounded in the constructivist pedagogical philosophy [25,26]. As argued, simply employing social computing and technological tools may not be enough to impact students’ learning positively [9,24]. Not surprisingly, AHSS and Business students shared the same sentiments with their peers in the Health Sciences and Medicine about the most beneficial tools. As the results show, the two groups’ thought online classes, assignment feedback, and video clips were the most helpful social computing software even though they ranked them differently. As the qualitative data suggests, students can learn from each other, share their thoughts on non-threatening platforms, and get feedback to enhance their learning [4,20]. In the same vein, the tools help students to have fun while learning using social computing software such as gamification and Bb Collaborate. In addition to indicating how students can reach them, online educators need to consider the added value that video, gamification, online discussion, assignment feedback, and synchronous online class can bring.

Reinforcing the necessity of social computing software and planning for effective teaching and learning in the course, students identified particular tools that educators need to consider in planning and delivering courses in the future. They advised that educators may want to consider announcement (on Bb), assignment feedback, online class (Bb Collaborate), and email as essential for effectively planning and delivering the course. This finding reiterates the need for communication and interaction between faculty and students to be taken seriously for effective teaching and learning [24]. The study’s findings also explain why students prefer some social computing software and tools over others–namely, because they foster interactions, help understand concepts, and are easy to use [2,24,25], as the qualitative data showed. Conversely, students thought some technological applications were less productive and more time-consuming, and less beneficial. These findings assist in explaining which key factors influence students’ negative and positive thoughts concerning the application of social computing tools in the educational process. Besides, the data reinforces the view that using technological tools does not automatically translate into effective teaching and learning [7], even though it is essential to assist students in building digital transferrable skills [6]. Educators, therefore, need to select technological tools that can easily be used by students to foster collaborative learning and assist them in making connections to real-life situations [2,4,25]. Doing this will ensure that students do not experience technological anxiety [9] and support their learning in line with their needs and preferences [8].

In selecting social computing and technological tools with the potential to enhance teaching and learning with minor issues, students recommend announcements, online discussion, gamification, chat-box, and email are highly essential. The finding underscores the importance of communication and collaboration in effective teaching and learning using social computing tools [5,24]. However, students’ preferences for the tools may differ based on their specializations. For instance, AHSS and Business students chose gamification as the fifth top software while Health Sciences and Medicine selected the online class. This result indicates that idiosyncrasies particular to student specializations’ may be a critical decision-making factor when selecting optimal social computing software to teach students across the university. So seeing a social computing tool as a size fits all may be counter-productive.

## Conclusion

Our findings reinforce the conclusions of previous studies indicating that social computing software and educational tools can indeed assist in fostering students’ learning, especially in situations with limited face-to-face interactions. However, educators leading students’ learning in courses with students from various backgrounds must understand that using some of these tools will be less attractive or beneficial. In particular, students taking university or college elective courses may have different preferences, which could be connected to their perceived specializations’ orientations and needs. As our data revealed, Arts and Social Sciences students shared some views and preferences on the relevance of social computing software with their contemporaries in the Medical and Science oriented disciplines. For instance, the two groups felt announcements, assignment feedback, online class, gamification, and email were helpful in their classes. However, the Arts and Social Sciences students recommended gamification, assignment feedback, chat box, announcement, and email. In contrast, the Medical students recommended announcements, online classes, assignment feedback, email, and chat box for future consideration.

Drawing on these findings, faculty need to provide customized and personalized learning experiences to enhance educational learning opportunities based on equity, differentiation, and inclusivity. In doing this, faculty may consider diagnostic feedback at the beginning of the semester to further determine what may support the objective of ensuring that all students learn as effectively as possible. Faculty could also conduct regular formative assessments to gain insights into students’ learning using different social computing tools. Using the critical incident questionnaire to garner feedback from learners during the semester would also help understand students’ preferences and identify tools most beneficial for their learning. As shown in the literature, CIQ assists facilitators in collecting data about critical incidents impacting students’ learning and the factors responsible for such events, thereby helping them make decisions for improving the learning process [4,40]. We contend that students will not be left behind when these measures are considered and applied in the learning process. By being involved in a consultation process, students are likely to be more engaged and feel empowered as they would not simply be passive participants in a static course paradigm but rather active contributors to their learning by providing feedback about desired modalities and digital tools. Moreover, students would potentially feel appreciated and valued, which may also positively impact their psychological well-being.

On their part, students should provide honest feedback when given the opportunity by the faculty, as that will assist in selecting and deploying the appropriate tools to support their learning. Besides, they should be willing to experiment with new learning tools and be open to learning from peers regarding how to better leverage new tools for learning through collaboration with others. In addition to faculty and student roles, university administration also needs to continuously provide opportunities for faculty and students to get oriented when new tools are introduced or purchased. Doing this will ensure that faculty and students can appreciate the latest tools’ potential and understand how to tap their capabilities for a more effective teaching and learning experience.

## Supporting information

S1 Raw data (XLSX)

S1 Appendix (DOCX)
