# Correction: Understanding student perceptions of social computing and online tools to enhance learning

**DOI:** 10.1371/journal.pone.0315521

**Published:** 2024-12-05

**Authors:** Semiyu Adejare Aderibigbe, Wafa Barhoumi Hamdi, Khadeegha Alzouebi, William Frick, Assad Asil Companioni

The first author’s name is spelled incorrectly. The correct name is: Semiyu Adejare Aderibigbe. The correct citation is: Aderibigbe SA, Hamdi WB, Alzouebi K, Frick W, Companioni AA (2022) Understanding student perceptions of social computing and online tools to enhance learning. PLOS ONE 17(10): e0276490. https://doi.org/10.1371/journal.pone.0276490
