# Supplementary material for: Understanding student perceptions of social computing and online tools to enhance learning
Source: PLoS One. 2022 Oct 27;17(10):e0276490. doi: 10.1371/journal.pone.0276490 (PMC9612442; doi:10.1371/journal.pone.0276490)
Supplement: S1 Appendix — (DOCX) [file pone.0276490.s002.docx]

**Appendix: Questionnaire on Understanding Student Perceptions of Social Computing and Online Tools to Enhance Learning**

Dear student,

It has been a pleasure to learn with you this semester. Without a doubt, we have learned from your experiences and contributions in the classes. As discussed, educators and professionals must keep exploring strategies to strengthen their professional practice. As such, we would like to know what worked in the current class and what needs to be considered for the effective use of social computing tools in the future.

We hope you would kindly take a couple of minutes to complete this questionnaire. Your response will be treated confidentially and solely used for the purpose of this study.

Please click on the box to indicate your willingness to participate in the study.

**Section A**: Select as applicable to you.

| Gender | M F |
| --- | --- |
| College | Arts, Humanities & Soc. Sc. Business Admin Communication  Computing & Informatics Dental Medicine Engineering  Fine Arts & Design Health Sciences Law  Medicine Pharmacy Sciences  Sharia & Islamic Studies |
| Year of Study | Year 1 Year 2 Year 3 Year 4 Year 5 |
| Age | 17-20 21-24 25-28 29-32 33 and above |

**Section B:** The tools listed in the table are used in this university elective course during the current COVID-19 time. Please select four tools you consider the most beneficial in the blended learning class.

| **Social Computing Tools** | **Currently Beneficial** |
| --- | --- |
| Online discussion forum |  |
| Blogs |  |
| Journals |  |
| Assignment feedback |  |
| Online class (through Collaborate) |  |
| Chat box in Collaborate |  |
| Icons in Collaborate, e.g. smiley face and thumb up |  |
| Video clips |  |
| Online class group discussion |  |
| Gamification (Kahoot Game) |  |
| Online office hour (through Collaborate) |  |
| Email |  |
| Phone |  |
| MS Team |  |
| Announcement (on Bb) |  |

Please provide at least two reasons for the choice of your selection above.

|  |
| --- |

**Section C:** To enhance teaching and learning in future classes in the COVID-19 context, facilitators and students need to prioritize some of the items below. Based on your experience, please rank the items in terms of importance. Consider one (1) as the most important and ten (10) as the least important.

| **Social Computing Tools** | **Future Preference Ranking** |
| --- | --- |
| Online discussion forum |  |
| Blogs |  |
| Journals |  |
| Assignment feedback |  |
| Online class (through Collaborate) |  |
| Chat box in Collaborate |  |
| Icons in Collaborate, e.g. smiley face and thumb up |  |
| Video clips |  |
| Online class group discussion |  |
| Gamification (Kahoot Game) |  |
| Online office hour (through Collaborate) |  |
| Email |  |
| Phone |  |
| MS Team |  |
| Announcement (on Bb) |  |

Please provide at least two reasons for the choice of your selection above.

|  |
| --- |

Thank you for taking the time to complete the survey and all the best in your future endeavors.
